# Facial Afro‐Caribbean Childhood Eruption Treated by Topical Erythromycin and Tretinoin

**DOI:** 10.1111/jocd.16646

**Published:** 2024-11-07

**Authors:** Nesrine Ben Salah, Mouna Korbi, Houda Ben Abdelwahed, Ines Lahouel, Samiha Mabrouk, Monia Youssef, Hichem Belhadjali, Jameleddine Zili

**Affiliations:** ^1^ Dermo‐Respiratory Research Laboratory LR20 SP 03, Dermatology Department, Fattouma Bourguiba Hospital University of Medicine Monastir Tunisia; ^2^ Anatomopathology Department, Fattouma Bourguiba Hospital University of Medicine Monastir Tunisia

To the Editor,

Facial Afro‐Caribbean childhood eruption (FACE), also known as childhood granulomatous periorificial dermatitis, is a rare and benign granulomatous condition first described by Gianotti et al. in 1970 [1]. This condition primarily affects dark‐skinned prepubescent children and should be differentiated from perioral dermatitis, sarcoidosis, granulomatous rosacea, and lupus miliaris disseminatus faciei. Here, we present the first case of FACE successfully treated with topical erythromycin and tretinoin (TRT), suggesting a new potential therapy for this condition.

A 16‐year‐old boy presented with a 3‐month history of yellowish, nonitchy micropapules around the mouth, nose, and upper and lower eyelids. He had no personal or family history of skin disorders and no history of atopy. The rash had not been preceded by the use of corticosteroids or other topical products. Physical examination revealed multiple monomorphic, lupoid, red‐to‐yellow papules ranging from 1 to 3 mm in diameter, accompanied by erythema and scaling (Figure 1a). There were no pustules or comedones. The rest of the skin and general physical examination were normal. Dermoscopic evaluation showed a yellow‐orange background with yellow‐white globules and white scales. Laboratory tests, including calcium level, conversion enzyme assay, and tuberculin reaction, were normal. Chest X‐ray and ophthalmological examination results were also normal. Histological examination of one of the perioral papules showed a diffuse granulomatous infiltrate in the dermis with histiocytes, multinucleated giant cells, and a heavy lymphocytic component. There was no caseation necrosis (Figure 2). No Demodex Folliculorum was observed, and special stains for fungi and acid‐fast bacilli were negative. These findings were consistent with a diagnosis of FACE. Standard patch testing, including European baseline and cosmetic allergens, showed no positive reactions. Treatment with topical erythromycin and tretinoin (*Erylik gel*: erythromycin 4%, tretinoin 0.025%) was initiated with one application daily. The patient responded well, showing improvement in skin lesions after 4 weeks (Figure 1b).

**FIGURE 1:**
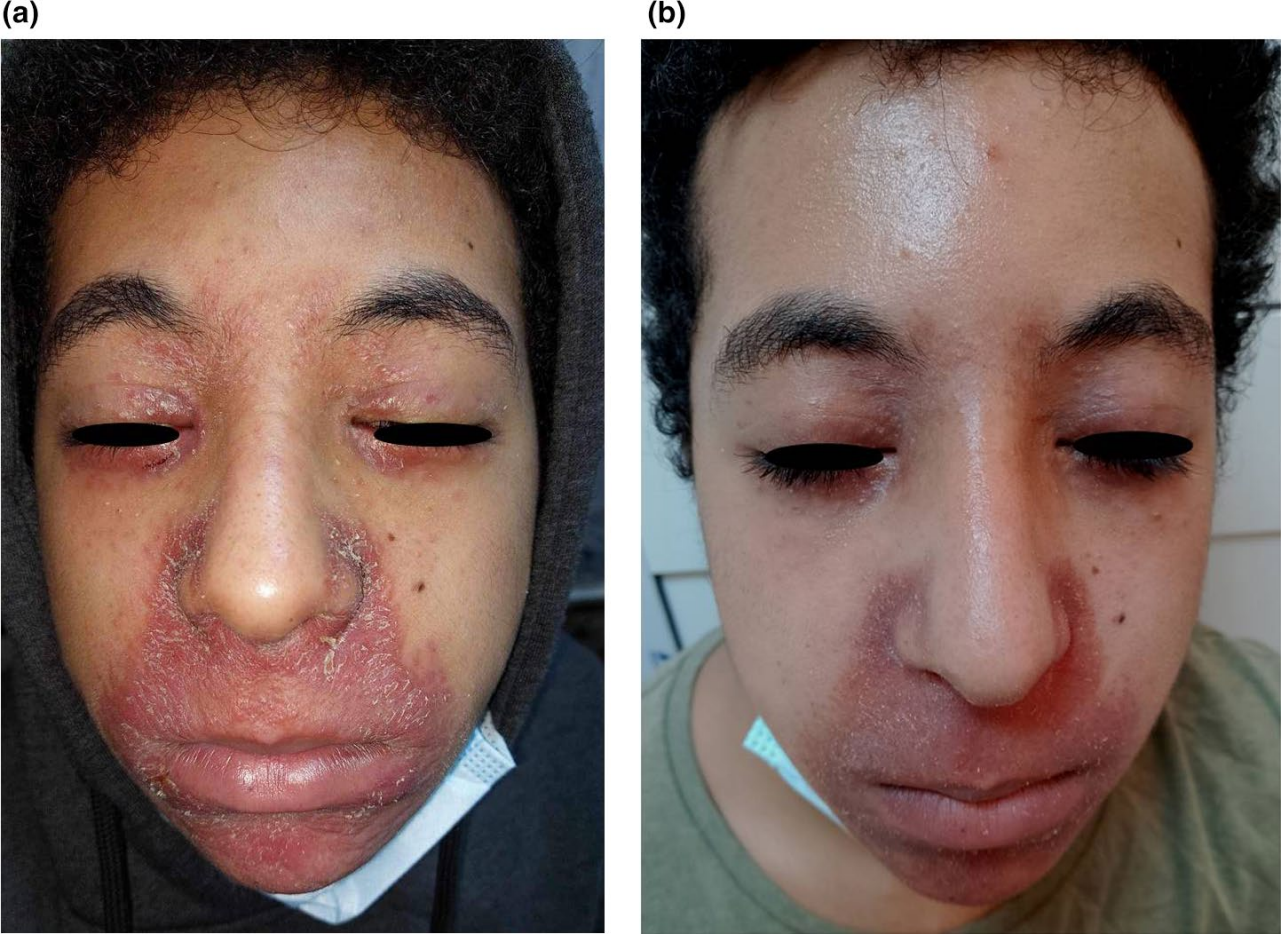
(a) Monomorphic and lupoid papules with erythema and scaling around the mouth, nose, and upper and lower eyelids. (b) Improvement of the skin lesions after 4 weeks of treatment with TRT.

**FIGURE 2:**
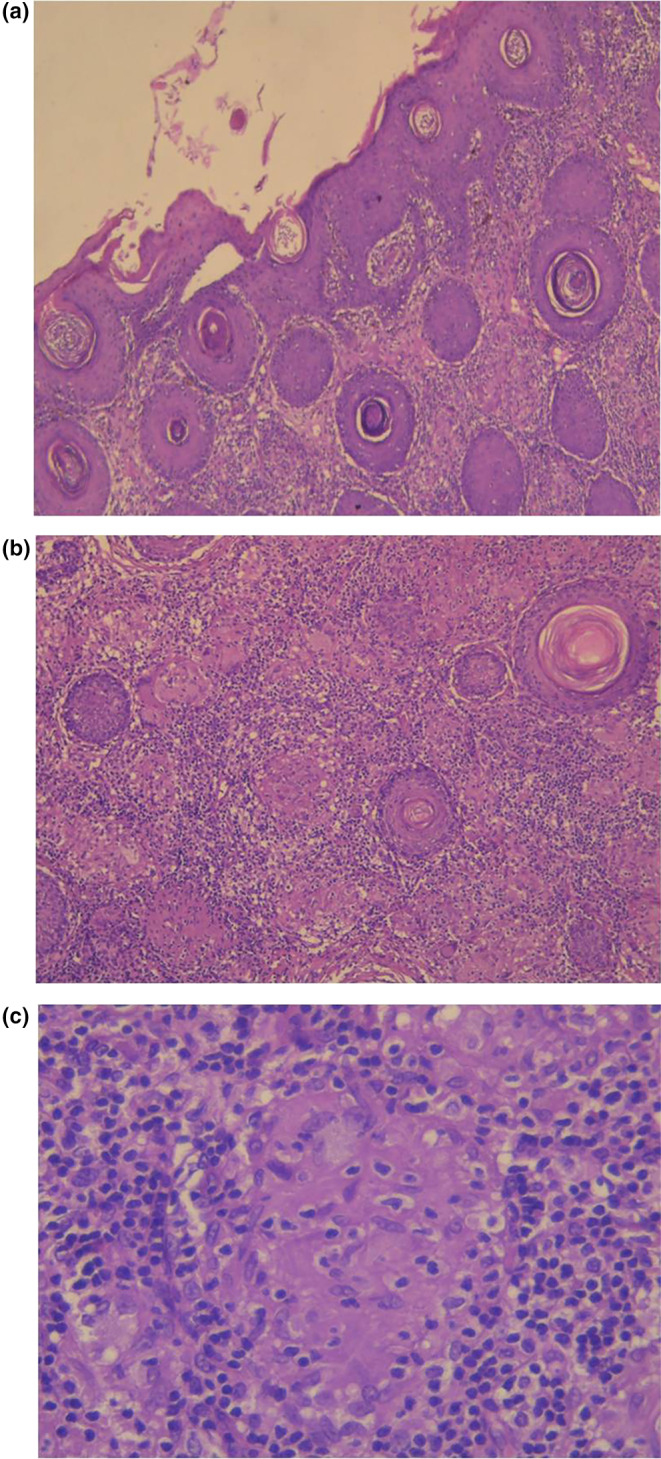
Histological examination revealed a diffuse granulomatous infiltrate of the dermis comprising histiocytes, multinucleated giant cells, and a heavy lymphocytic component. (a: H and E stain, ×40). (b: H and E stain, ×100). (c: H and E stain, ×400).

FACE is a rare, benign condition that presents as small, monomorphic papular eruptions around the mouth, nose, and eyes, without pustules, comedones, or scarring [2]. Histologically, it features nonspecific perifollicular granulomatous inflammation [2]. The etiology remains unknown, but some reports suggest associations with allergens or irritants such as bubble gum, formaldehyde, cosmetic preparations, and antiseptic solutions [3]. Long‐term use of topical steroids can induce or worsen FACE [3]. The condition, which primarily affects prepubertal children with darker skin types, tends to resolve spontaneously over several months without scarring. Management typically involves discontinuing topical corticosteroids [4, 5]. Although FACE is self‐limiting, treatment aims to reduce the duration of the condition. Oral tetracyclines, minocycline, doxycycline, erythromycin, and metronidazole have shown good results, as have topical treatments like metronidazole, pimecrolimus, and tacrolimus. Topical agents combined with oral treatments, including adapalene, clindamycin, azelaic acid, and photodynamic therapy, may also be effective [4, 5]. Oral isotretinoin may be considered for persistent cases. To date, there have been no reports of TRT as a treatment for FACE [6].

Patients and their families should be reassured that FACE is benign and self‐limited. We propose that TRT may be considered an effective first‐line treatment for FACE in children. Further research is needed to confirm its efficacy and establish optimal dosing and duration.

## Author Contributions

N.B.S., I.L., M.K., and H.B.A. performed the research and contributed essential reagents or tools. N.B.S., I.L., M.K., H.B.A., H.B., and J.Z. analyzed the data. N.B.S., M.K., and J.Z. wrote the paper. S.M. and M.Y. performed the research and analyzed the data.

## Consent

Written informed consent was obtained from the parent's patient to publish this report in accordance with the journal's patient consent policy.

## Conflicts of Interest

The authors declare no conflicts of interest.

## Data Availability

Data sharing is not applicable—no new data generated.
